# Identification of Regulatory Functions of LncRNAs Associated With *T. circumcincta* Infection in Adult Sheep

**DOI:** 10.3389/fgene.2021.685341

**Published:** 2021-06-14

**Authors:** Praveen Krishna Chitneedi, Rosemarie Weikard, Juan J. Arranz, María Martínez-Valladares, Christa Kuehn, Beatriz Gutiérrez-Gil

**Affiliations:** ^1^Institute of Genome Biology, Leibniz Institute for Farm Animal Biology (FBN), Dummerstorf, Germany; ^2^Departamento de Producción Animal, Facultad de Veterinaria, Universidad de León, León, Spain; ^3^Departamento de Sanidad Animal, Facultad de Veterinaria, Universidad de León, León, Spain; ^4^Instituto de Ganadería de Montaña, CSIC-Universidad de León, León, Spain; ^5^Faculty of Agricultural and Environmental Sciences, University of Rostock, Rostock, Germany

**Keywords:** adult sheep, gastrointestinal infection, nematode, abomasal lymph node, long non-coding RNA, functional annotation, gene co-expression, pathways

## Abstract

Several recent studies have demonstrated the role of long non-coding RNAs (lncRNAs) in regulating the defense mechanism against parasite infections, but no studies are available that investigated their relevance for immune response to nematode infection in sheep. Thus, the aim of the current study was to (i) detect putative lncRNAs that are expressed in the abomasal lymph node of adult sheep after an experimental infection with the gastrointestinal nematode (GIN) *Teladorsagia circumcincta* and (ii) to elucidate their potential functional role associated with the differential host immune response. We hypothesized that putative lncRNAs differentially expressed (DE) between samples from animals that differ in resistance to infection may play a significant regulatory role in response to nematode infection in adult sheep. To obtain further support for our hypothesis, we performed co-expression and functional gene enrichment analyses with the differentially expressed lncRNAs (DE lncRNAs). In a conservative approach, we included for this predictive analysis only those lncRNAs that are confirmed and supported by documentation of expression in gastrointestinal tissues in the current sheep gene atlas. We identified 9,105 putative lncRNA transcripts corresponding to 7,124 gene loci. Of these, 457 were differentially expressed lncRNA loci (DELs) with 683 lncRNA transcripts. Based on a gene co-expression analysis via weighted gene co-expression network analysis, 12 gene network modules (GNMs) were found significantly correlated with at least one of 10 selected target DE lncRNAs. Based on the principle of “guilt-by-association,” the DE genes from each of the three most significantly correlated GNMs were subjected to a gene enrichment analysis. The significant pathways associated with DE lncRNAs included ERK5 Signaling, SAPK/JNK Signaling, RhoGDI Signaling, EIF2 Signaling, Regulation of eIF4 and p70S6K Signaling and Oxidative Phosphorylation pathways. They belong to signaling pathway categories like Cellular Growth, Proliferation and Development, Cellular Stress and Injury, Intracellular and Second Messenger Signaling and Apoptosis. Overall, this lncRNA study conducted in adult sheep after GIN infection provided first insights into the potential functional role of lncRNAs in the differential host response to nematode infection.

## Introduction

The central dogma of molecular biology states that DNA is transcribed into mRNA and mRNA is translated into protein products (Crick, 1970). There is an increasing interest, however, also in the non-protein coding RNAs (ncRNAs), which are estimated to comprise up to 80% of the entire transcriptome in mammals (The ENCODE Project Consortium, 2012; Hon et al., 2017; Srijyothi et al., 2018). The ncRNAs are classified into several sub-classes, such as small non-coding RNAs, including microRNAs and small interfering RNAs, processed small RNAs (Wilusz et al., 2009), transcription start site–associated RNAs (Seila et al., 2008), promoter associated RNAs (PARs) (Taft et al., 2009), enhancer RNAs (eRNAs) (Kim et al., 2015) and long non-coding RNAs (lncRNAs) (Wilusz et al., 2009). LncRNAs are defined as non-coding RNA transcripts with a length greater than 200 nucleotides. Although lncRNAs are biochemically resembling mRNAs, they generally do not encode protein products (Ponting et al., 2009). LncRNAs are divided into several subgroups according to the positional genomic relationship between lncRNAs and their neighboring protein-coding genes including overlapping, antisense, intronic and intergenic lncRNAs. Advances in computational biology and evolution of sensitive RNA sequencing and epigenomic technologies have facilitated the discovery of numerous lncRNAs and encouraged the study of their functional roles.

Based on their potential function, lncRNAs can be broadly classified into three main categories: (1) lncRNA transcripts, which are non-functional; (2) lncRNAs as regulators of transcription, which act as *cis*- and *trans*-active modulators of protein-coding gene expression (Kopp and Mendell, 2018), and (3) lncRNA transcripts involved in post-transcriptional regulation, which includes alternative mRNA splicing regulation, translational control and competing with regulatory endogenous RNAs (Riquelme et al., 2016; Dykes and Emanueli, 2017). It has been found that lncRNAs exhibit splice junctions and introns (Hiller et al., 2009) and cell- and tissue- specific expression patterns. The misexpression of lncRNAs has been shown to contribute to neurological disorders, cancer (Qureshi et al., 2010), susceptibility to infection, metabolic disorders such as diabetes and obesity (Moran et al., 2012; Zhao and Lin, 2015), and other diseases (Wapinski and Chang, 2011). In addition to their identification and cataloging, the functional annotation of the discovered lncRNAs is also challenging, as they are sparsely and cell type-specifically expressed, which might limit their function to few biological states (Derrien et al., 2012). Furthermore, at sequence level lncRNAs are poorly conserved across species (Ulitsky and Bartel, 2013; Hezroni et al., 2015).

The functional annotation of lncRNAs aims to identify or predict the possible biological process with which an lncRNA transcript can be interrelated, its putative mechanism of action, potential interacting partners and putative functional elements within the RNA locus (Cao et al., 2018). The annotation catalog of lncRNAs is far from complete even in humans and mice, and much work remains to be done in the field of lncRNAs annotation in the genomes of livestock species (Weikard et al., 2017). Currently, several lncRNA databases are available (Maracaja-Coutinho et al., 2019), which provide annotations of lncRNAs, mostly referring to the human genome. Among them, the NON-CODE database (Zhao et al., 2016) currently offers a comprehensive collection of lncRNA transcripts that have been experimentally confirmed in humans and 15 animal species (including the farm animals cow, pig, and chicken, but not sheep). With regard to the sheep genome, although there are many predicted protein coding genes known, only 30% of them are carrying an Ensembl identifier in the OAR v3.1 reference genome [Ensembl annotation based on adult Texel sheep (Clark et al., 2017)]. Most of the detected and characterized lncRNAs of the sheep genome have been annotated through the sheep atlas project based on the Texel breed (Clark et al., 2017).

Recently, another sheep reference genome Oar rambouillet v1.0 (GCA_002742125) has become available, providing a highly contiguous sheep genome with an annotation and mapping of transcription start sites (Salavati et al., 2020). However, this new reference genome contains a similar number of coding and non-coding genes compared to the OAR v3.1 reference genome. Currently, the Ensembl database contains 1,858 and 2,236 putative lncRNAs in the OAR v3.1 and the OAR rambouillet v1.0 genome annotation, respectively^1^ (Oar rambouillet v1.0^2^).

LncRNAs are known to act as key regulators of the immune response by a variety of mechanisms (Chen et al., 2017; Menard et al., 2019). Some recent lncRNA studies conducted in humans (Rochet et al., 2019) and mice (Menard et al., 2018) have demonstrated the role of lncRNAs in regulating the defense mechanisms against parasite infections. However, up to now no sheep-specific studies are available that investigated the importance of lncRNAs for the immune response to nematode or parasite infection.

Gastrointestinal parasite infections in sheep are a major challenge to sheep husbandry due to great economic losses, impairment of animal welfare and difficulties in appropriate treatment and prophylaxis under the dominating extensive pasture system in sheep production (Roeber et al., 2013; Ruano et al., 2019). The aim of the current study was therefore to detect lncRNAs in the transcriptome of abomasal lymph node (ALN) tissue samples extracted from adult Spanish Churra sheep after an experimental infection with the gastrointestinal nematode (GIN) *Teladorsagia circumcincta* and to elucidate their potential functional role associated with the host immune response against GIN infection in adult sheep. The current study is based on the OAR v3.1 genome annotation, as it was the most widely used annotation and was also used as reference annotation in our previous RNA-sequencing (RNA-seq) studies (Chitneedi et al., 2018, 2020), which were conducted with the same samples as the current study.

We hypothesized that putative lncRNAs differentially expressed between samples from animals that differ in infection resistance may play a significant regulatory role in response to nematode infection in adult sheep. To validate our hypothesis, we performed co-expression and functional gene enrichment analyses with selected lncRNAs that (i) were differentially expressed in our study in response to GIN infection, (ii) are included in the current sheep gene atlas, and (iii) showed expression in gastrointestinal and lymph node tissue (Clark et al., 2017; Bush et al., 2018). Thus, this work will contribute to the aim of the global network for Functional Annotation of Animal Genomes (FAANG) to provide high quality functional annotations of farm animal genomes (Andersson et al., 2015).

## Materials and Methods

### Experimental Design and RNA Sequencing

The experiment and the criteria for animal selection have been described earlier in detail in Chitneedi et al. (2018). The whole experiment was carried out according to the current National Spanish legislation on the protection of animals used in experimentation (Royal Decree 53/2013) and the approval of the competent body of the regional government, Junta de Castilla y León (ULE_024_2015). Initially, a commercial flock of the Churra dairy sheep breed including 119 dairy ewes, raised under a commercial, semi-intensive management system representing natural GIN infection conditions was sampled to measure the fecal egg count (FEC). Based on the distribution of FEC values in the selected flock [phenotypic values: average = 25.59 eggs per gram (epg); *SD* = 60.31 epg], we preselected a group of 10 animals showing extremely low FEC values (range 0–15 epg) and a group of eight animals showing very high FEC values (range 90–225 epg). These animals were transferred to the experimental farm of the Mountain Livestock Institute (IGM, León, Spain), where they were exposed to a first standardized experimental infection (EI1) with *T. circumcincta* third stage larvae (L3) after an antihelmintic treatment (oral dose of ivermectin). *T. circumcincta* is one of the most common GIN parasites that infects different sheep breeds, in particular with a high incidence in Churra sheep (Diez-Banos et al., 1992; Castilla-Gomez De Aguero et al., 2020). Based on the accumulative FEC at days 14–31 after the first experimental infection (EI1), six ewes were classified as ‘susceptible’ (SUS, range: 2,310–9,666 epg; average: 5,594 ± 2,661 epg) and six ewes as ‘resistant’ (RES, range: 0–915 epg; average: 308 ± 338 epg). After a second experimental infection (EI2), these 12 animals were sacrificed at day 7 after infection, and abomasal lymph node (ALN) tissue samples were collected. Total RNA extracted from these samples was used for library preparation using the KAPA Stranded mRNA-Sequencing Kit that starts with an oligo dT selection step (Roche). RNA sequencing (RNA-seq) using an Illumina Hi-Seq 2000 platform generated ‘paired-end’ reads of 75 bp, with a sequencing depth of 30 M fragments per sample. The FASTQ format data from the 12 ALN samples were subjected to the subsequent bioinformatics workflow summarized in Supplementary Figure 1.

### Alignment and Transcript Assembly

The script including the specific parameters used for different tools included in the bioinformatic workflow is provided in Supplementary File 1. Initially, potential adapter sequences were removed from RNA reads with *cutadapt_v1.18* (Martin, 2011) followed by trimming of poor-quality sequences from reads using *quality trim v1.6.0* (Robinson, 2015) to improve subsequent read alignment. After trimming, the reads were aligned against the *Ovis aries* reference genome Oar_v3.1 (GCA_000298735.1) with Ensembl annotation release 95^3^ using the alignment tool *HISAT2 v2.1.0* (Pertea et al., 2015). The aligned unsorted reads were sorted using *samtools v1.9* (Li, 2011). The aligned and sorted read data were assembled into transcripts using *StringTie v1.3.5* (Pertea et al., 2015) and the *O. aries* reference genome Oar_v3.1 (GCA_000298735.1) with Ensembl annotation release 95 (see Text Footnote 3) for a reference-guided transcriptome assembly approach (Pertea et al., 2015). This concept of reference-guided transcriptome assembly, which depends on a genome assembly of the target organism, takes benefit from existing information about transcribed genome elements, but enables detection of previously unknown transcripts. This is of particular importance for livestock lncRNA investigation, because the catalog of lncRNAs is by far not complete up to now. *StringTie* starts by building clusters from reads aligned to the genome and then creates a splice graph to identify transcripts (Pertea et al., 2015). In addition to tagging the unannotated genes, *StringTie* also tags those genes provided in the reference genome annotation. After assembling the reads from all 12 samples individually, the full set of transcript assemblies was passed to *StringTie*’s merge function, which merges the gene structures found in any one of the samples. As in some samples only partially covered transcripts were assembled in the initial *StringTie* run, the merging step created a set of transcripts that is consistent across all samples, so that the transcripts can be compared in the subsequent steps (Pertea et al., 2015). Additionally, the merged annotation file was compared with the reference annotation Ensembl release 95 (see Text Footnote 3) of the OAR_v3.1 genome using *gffcompare v-0.10.6* (Pertea and Pertea, 2020) to identify novel transcripts and to determine how many assembled transcripts were fully or partially consistent with annotated genes.

### LncRNA Detection

We used the lncRNA prediction tool *FEELnc* (Wucher et al., 2017) to indicate lncRNAs in the newly established merged annotation file of the ALN transcriptome in our sheep samples. The selection of this software was based on a previous study of our research group comparing different bioinformatic tools for lncRNA prediction (Weikard et al., 2018). The *FEELnc* analysis includes three steps: (i) filtering of candidate lncRNA transcripts; (ii) exploring coding potential and nucleotide composition of candidate lncRNA transcripts; and (iii) classification of the finally predicted lncRNA transcripts. In the first step, protein-coding genes, monoexonic transcripts (except for those in antisense direction to a coding gene) and transcripts with a size less than 200 nt were excluded, and a file was generated comprising potential candidate lncRNA transcripts. In the second step, the coding potential score was determined for each of the candidate lncRNA transcripts by considering absence of an open reading frame and *k*-mer nucleotide composition using the shuffle mode option of *FEELnc*. Finally, those putative lncRNAs with evidence for non-coding characteristics were classified with regard to their localization and their direction of transcription compared to adjacent RNA transcripts (protein-coding and non-coding loci). This classification of putative lncRNAs with respect to associated mRNAs or protein-coding genes (or other ncRNAs) might provide initial indication on potential lncRNA functions. In addition, to evaluate, if the putative lncRNA transcripts detected in the ALN sheep transcriptome were novel compared to the OAR_v3.1-r 95 annotation, the predicted lncRNAs were classified using the *gffcompare -r* option, and class code “u” assignment was taken as indication of a novel lncRNA.

### Read Count and Differential Gene Expression Analysis

**FIGURE 1:**
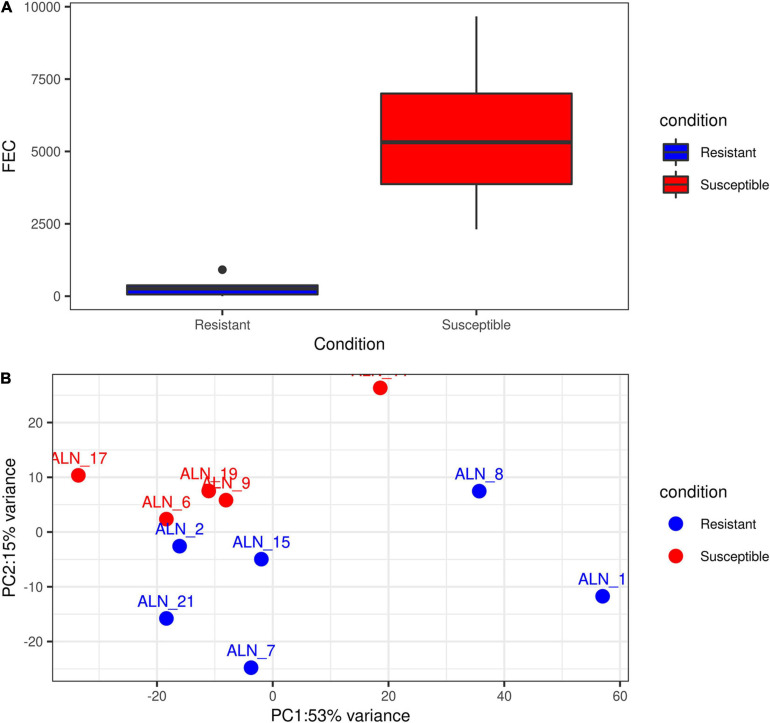
Exploratory plots of GIN infected resistant and susceptible sheep. **(A)** Distribution of accumulated fecal egg count (FEC) in resistant and susceptible sheep after a second experimental infection with GIN *Teladorsagia circumcincta.* Blue box represents the group mean of the resistant group and red box represents the group mean of the susceptible group. **(B)** PCA performed with the read count matrix of the 11 ALN RNA-seq datasets finally included in the differential gene expression analysis specifying the sample condition [resistant (6) and susceptible (5)].

The ‘*featureCounts*’ option of the package *subread v 1.6.3* (Liao et al., 2013) was used with the newly generated merged annotation file to calculate read count matrices for all samples and all annotated loci. Prior to differential gene expression (DGE) analysis of the samples, we plotted the accumulated phenotypic FEC data of the animals and also performed an exploratory Principal Component Analysis (PCA) of the sample transcriptomes for the initial six resistant and six susceptible animals (Supplementary Figure 2). The phenotypic information available for sample ALN_14 (Supplementary Figure 2A) showed that the accumulated FEC value of the respective animal after EI1 was the lowest among the susceptible samples. Considering the first two principle components (PC1 and PC2) of the transcriptome expression data, after excluding the ALN_14 this sample originally allocated into the susceptible group could not be clearly assigned to this group (Supplementary Figure 2B). Thus, in order to perform the subsequent analysis on two groups with clearly distinct phenotypes (Figures 1A,B), sample ALN_14 was considered as an outlier and was excluded from the subsequent DGE analysis. This DGE analysis was performed with the *DESeq2* R package (Love et al., 2014) to identify differentially expressed (DE) loci (coding and non-coding) between the two animal groups differing in susceptibility to GIN infection. Loci were considered significantly DE at a FDR < 0.05 after Benjamini–Hochberg correction for multiple testing.

### Gene Co-expression Analysis

For a first indication on the potential functional role of lncRNA highlighted as DE in the differential expression analysis, we performed co-expression analyses. To this end, we applied the R package for Weighted gene co-expression network analysis (*WGCNA, v1.64*) (Langfelder and Horvath, 2008). This method was implemented using the parameters mentioned in Weikard et al. (2018). Briefly, WGCNA was performed initially with all the protein-coding and non-coding genes expressed in the 11 sheep ALN samples serving as expression data input except for those genes serving as target variables. We selected 10 lncRNAs as target variables for the WGCNA according to the following criteria: (i) they displayed significantly differential expression between the RES and SUS sheep in response to GIN infection, (ii) they had been reported previously as expressed in gastrointestinal and lymph node tissue in the sheep gene atlas study (Clark et al., 2017) and had been predicted as lncRNAs in the sheep lncRNA study linked to the sheep gene atlas study (Bush et al., 2018), (iii) finally, they displayed a clear exon-intron structure as verified by visual inspection of reads aligned to the selected DE lncRNA transcripts using the Interactive Genomics Viewer (IGV) (Thorvaldsdottir et al., 2013).

The co-expressed genes within the WGCNA were identified as gene network modules (GNMs) marked with different colors. We assumed that highly interconnected genes within a GNM are co-regulated and might be involved in similar biological pathways. Those GNMs that were highly significantly correlated with at least one DE lncRNA transcript (*r* > | 0.75| and *p* < 0.01) were selected for pathway enrichment analysis of the included DE genes to obtain indication on a potential functional role of the correlated DE lncRNA.

### Enrichment and Pathway Analysis

To predict the potential biological function of the DE lncRNAs and associated biological pathways, we performed the gene set enrichment pathway analysis using the Qiagen Ingenuity analysis package (IPA^4^) with the DE genes from those GNM that were correlated with a specific lncRNA. Only annotated protein-coding genes in significantly correlated GNMs were included in the IPA analysis, whereas functionally unannotated and non-protein-coding genes were excluded.

## Results

### Alignment and Transcriptome Assembly

After trimming of adapters, primers and poor-quality sequences, the data from the 12 samples initially included in the transcriptomic study comprised 30 to 50 M fragments per sample, which were subjected to further analysis. An average of 89.8% of the reads were aligned against the OAR_v3.1 reference genome. Finally, after performing the reference-guided transcriptome assembly, the final new annotation file after merging information across all 12 samples comprised 77,039 transcripts. These transcripts corresponded to a total of 44,203 transcribed gene loci, which is an average of 1.74 isoform transcripts per gene locus.

### LncRNA Detection and Classification

After filtering out protein-coding transcripts, based on their coding potential score and nucleotide composition, the *FEELnc* program identified 9,105 lncRNA transcripts corresponding to 7,124 putative lncRNA loci. This corresponds to 1.28 lncRNA transcripts per lncRNA locus, which is lower than the average number of transcript isoforms across all gene loci. We observed an equal strand distribution of these lncRNA transcripts with 50.2% (3,580 lncRNA loci) identified on the plus strand and 49.7% (3,544 lncRNA loci) on the minus strand. The average number of exons per lncRNA locus was 3.3 (median 2) with a mean exon length of 432.2 nt (median149 nt). The average number of exons per non-lncRNA locus was 14.3 (median 2) with a mean exon length of 289.8 nt (median 130 nt).

**FIGURE 2:**
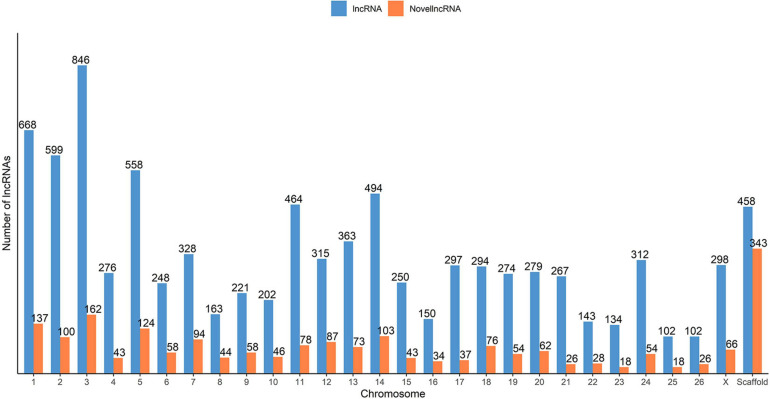
Distribution of the predicted putative lncRNAs across the sheep genome from the merged ALN RNA-seq datasets of the 12 ALN sheep transcriptome samples under study.

To classify the identified lncRNA transcripts, we used the sliding window size of 10,000 to 100,000 nt to check for the possible overlap with the nearest transcript from the reference annotation. In total, we identified a total of 19,162 potential predicted spatial lncRNA interactions between 8,676 lncRNA transcripts (originating from 6,854 lncRNA loci) and other loci in the merged annotation. Out of these, 11,250 were intragenic interactions, called as ‘genic’ by the *FEELnc* classifier, and 7,912 were intergenic. No potential spatial interaction was predicted for the remaining 429 lncRNA transcripts (270 lncRNA loci). Some of the lncRNA transcripts interacted with both genic and intergenic regions of different partner transcripts. In total, 3,004 lncRNA transcripts were in sense direction with potentially interacting genic regions and 5,216 lncRNA transcripts in antisense direction. In case of intergenic regions, 3,143 lncRNA transcripts were predicted in sense direction and 2,758 in antisense direction. The mean expression of the 7,124 lncRNA loci was 60.6 FPKM, with a median of 9.13 FPKM and in case of the rest 37,079 non-lncRNA gene loci, the mean expression was 12.9 FPKM (median 0.88). Of the total of 9,105 lncRNA transcripts found in our analysis (7,124 lncRNA loci), 2,092 lncRNA transcripts (1,393 lncRNA loci) were categorized as novel by gffcompare. The distribution of novel and known lncRNA transcripts across the ovine chromosomes is shown in Figure 2.

### Differential Gene Expression Analysis

After excluding the outlier sample (ALN_14) detected through the PCA and evaluation of phenotypic FEC values (Figures 1A,B), a total of 3,148 differentially expressed loci (DELs, protein-coding and non-coding) were detected at a significance threshold of *q* < 0.05 between the ALN transcriptomes of the RES and SUS sheep groups in response to GIN infection. Of these, 1,635 DELs were higher and 1,513 DELs were lower expressed after GIN infection in RES sheep compared to the SUS group. The list of DELs is shown in Supplementary Table 1. When investigating the 3,148 DELs, we found 683 lncRNA transcripts in 457 DELs. Of these, 263 lncRNA transcripts associated with 153 DELs were considered novel. The list of DE lncRNAs including the novel DE lncRNAs is provided in Supplementary Table 1.

### Gene Co-expression and Enrichment Analysis

**FIGURE 3:**
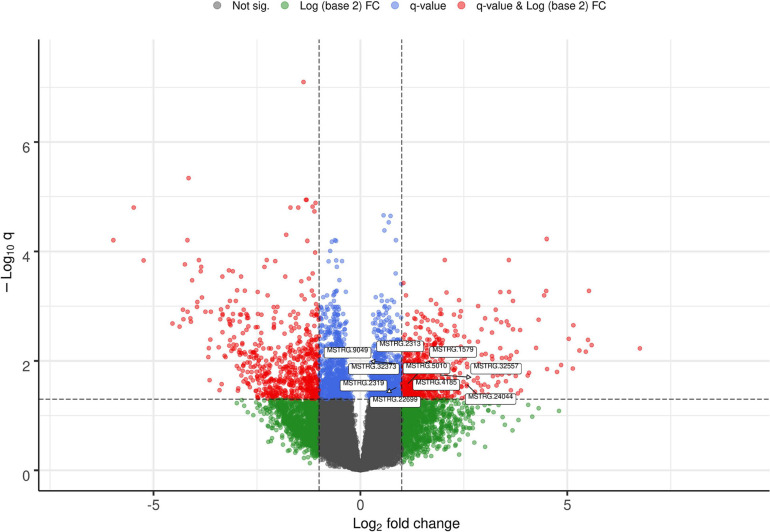
Volcano plot showing the differentially expressed (DE) genes with the selected target lncRNA loci for co-expression analysis.

**FIGURE 4:**
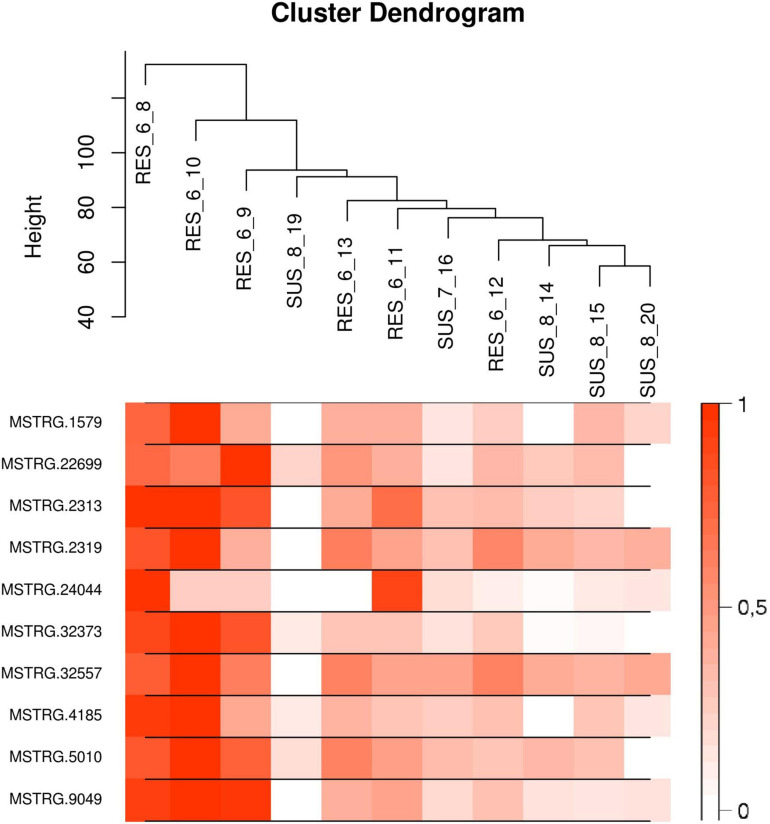
**(Top)** Cluster dendrogram plot of the ALN RNA-seq data to differentiate the resistant and susceptible sheep. **(Bottom)** Corresponding heatmap with the expression levels from the 10 selected lncRNAs showing a notable expression differentiation in resistant compared to susceptible samples.

The list of the 10 DE lncRNA loci selected for this analysis is shown in Table 1 and Figure 3 depicts results of the DE analysis for those 10 target DE lncRNA loci. The cluster dendrogram plot of the ALN transcriptome data from the RES and SUS sheep and the corresponding heatmap (Figure 4) show that the expression level of the 10 selected lncRNAs displayed a different pattern between the groups of differential resistance to GIN infection (Figure 4). Based on the module co-expression analysis with the 10 target lncRNAs, we found 88 GNMs (Supplementary Figure 3). Of them, 12 GNMs were significantly correlated (*p* < 0.01) with at least one of the 10 target lncRNAs included in the analysis (see Figure 4). Furthermore, we found that most lncRNAs were highly co-expressed with several GNMs at *r* > | 0.75| and *p* < 0.01.

**TABLE 1:** Differentially expressed (DE) lncRNAs selected for gene co-expression analysis.

**LncRNA**	**Chromosomal location (chr:start-end:strand, sense)**	**Overlapping gene**	**Classification of the identified lncRNA transcripts expressed in sheep abomasal lymph node relative to the overlapping gene^1^**	**Overlapping region from previous studies (chr:start-end:strand, sense)**	**Expression of overlapping gene regions from previous sheep studies^2^**
MSTRG.2313	1:188445304–188449367:+	*ENSOARG00000020259 (RPL35A)*	Intronic, antisense, class code - *S*	1:188445366–188448530:−	Prescapular lymph node in TxBF adult^3^
MSTRG.9049	14:50414613–50422485: +	*RPS19*	Intronic, antisense, class code - *S*	14:50414657–50422495: −	Prescapular lymph node in TxBF adult^3^
MSTRG.32373	9:36111002–36112522: +	*RPS20*	Intronic, antisense, class code - *S*	9:36111034–36112627: −	Prescapular lymph node in TxBF adult^3^
MSTRG.1579	1:109258493–109270297: +	*ENSOARG00000025559 (lincRNA located near IGSF9 and TAGLN2)*	Complete, exact intronic match, class code -=	1:109251911–109274557: +	Not calculated^4^
	1:109264963–109270297: +		Multi-exonic with at least one junction match, class code – *j*	1:109267606–109271242: −	Abomasum in Texel lamb/6–10 months^3^
MSTRG.2319	1:188856696–188859141: −	*Located between MUC20 and TNK2*	Novel (unknown, intergenic), class code – *u*	1:188856349–188859135: +	Hippocampus in TxBF adult^4^
	1:188856697–188859104: −		Novel (unknown, intergenic), class code – *u*		
	1:188856697–188859111: −		Novel (unknown, intergenic), class code – *u*		
	1:188856697–188859118: −		Novel (unknown, intergenic), class code – *u*		
	1:188856697–188859141: −		Novel (unknown, intergenic), class code – *u*		
	1:188856701–188859137: −		Novel (unknown, intergenic), class code – *u*		
MSTRG.5010	11:39016413–39020308: +	*ENSOARG00000010029 (RPL23)*	Intronic, antisense, class code - *S*	11:39016458–39020292: −	Omasum in TxBF lamb/new born^3^
MSTRG.4185	11:14061262–14065789: −	*ENSOARG00000004831 (CCL14)*	Intronic, antisense, class code - *S*	11:14061328–14065335: +	Omentum in Texel adult^3^
MSTRG.22699	3:10979130–10983453: −	*ENSOARG00000013275 (RPL35)*	Intronic, antisense, class code - *S*	3:10979613–10983240: +	Rumen in TxBF lamb/new born^3^
MSTRG.24044	3:133375792–133383230: −	*KRT8*	Intronic, antisense, class code - *S*	3:133375727–133383019: +	Rectum in Texel adult^3^
MSTRG.32557	9:57536379–57541099: +	*FABP4*	Intronic, antisense, class code - *S*	9:57536525–57541042: −	Omentum in Texel adult^3^

**^1^The classification of transcripts is based on the annotation tool Gffcompare -r option. ^2^TxBF adult refers to adult Texel × Scottish Blackface sheep. ^3^Clark et al. (2017)^4^Bush et al. (2018).**

For canonical biological pathway analysis, out of the 12 GNMs significantly correlated with at least one target lncRNA, we selected the DE genes from the three most highly significantly correlated GNMs: green, turquoise and yellow. Although other modules were also significantly co-expressed with the 10 selected lncRNAs, the genes from the other significantly correlated GNMs did not satisfy the criteria that were used to select the genes for enrichment pathway analysis. The blue and darkseagreen4 GNMs did not comprise DE genes and the other modules contained only a single or no protein-coding DE gene(s), which precluded biological pathway analysis. As shown in Table 2, Ingenuity pathway analysis revealed that the canonical pathways *ERK5 (Extracellular signal-related kinase) Signaling*, *SAPK/JNK (stress-activated protein kinases) Signaling, RhoGDI Signaling*, *EIF2 (eukaryotic translation initiation factors) Signaling*, *Regulation of eIF4 (Eukaryotic translation initiation factor 4) and p70S6K Signaling* and *Oxidative Phosphorylation* were significantly enriched by genes from GNMs green, turquoise and yellow. Table 2 also contains the list of DE genes within the enriched pathways for the three GNMs significantly correlated to at least one target lncRNA.

**TABLE 2:** Significant gene modules and canonical pathways associated with differentially expressed (DE) lncRNAs.

**GNM**	**Gene number**^1^	**Co-expressed DE lncRNAs**	**Correlation coefficient (lncRNA- GNM)**^2^	**Top canonical pathways associated with GNM**	**−log(*p*-value)**	***z*-score**	**Genes involved**
Green	303	MSTRG.1579 MSTRG.2313 MSTRG.2319 MSTRG.32373 MSTRG.32557 MSTRG.5010 MSTRG.9049	−0.76 −0.85 −0.84 −0.8 −0.88 −0.93 −0.84	ERK5 signaling	4.55	−2.45	*RAP2A, PTPN11, GNAQ, KRAS, GNA13, ELK4*
				SAPK/JNK signaling	3.44	−2.45	*RAP2A, PTPN11, MAP3K13, MAP3K7, KRAS, GNA13*
				3-phosphoinositide degradation	3.4	−1.89	*SYNJ2, PTPN11, NUDT9, PIKFYVE, PPP1CA, RNGTT, PPFIA4*
Yellow	505	MSTRG.1579 MSTRG.2313 MSTRG.2319 MSTRG.32373 MSTRG.32557 MSTRG.4185 MSTRG.5010 MSTRG.9049	−0.92 −0.82 −0.89 −0.86 −0.85 −0.97 −0.8 −0.83	RhoGDI signaling	3.96	2.33	*ROCK1, ROCK2, ITGA3, PAK1, CFL1, CDH6, ARHGDIA, ARHGEF10, DLC1, GNG12*
				Axonal guidance signaling	2.77	NaN	*GLI2, CFL1, ADAM15, BCAR1, SEMA6C, ROCK1, ROCK2, ITGA3, PAK1, PLCB4, MMP11, PLXNB2, SEMA3C, PLCL1, GNG12*
Turquoise	1517	MSTRG.1579 MSTRG.2313 MSTRG.2319 MSTRG.32373 MSTRG.32557 MSTRG.4185 MSTRG.5010 MSTRG.9049	0.92 0.9 0.94 0.88 0.93 0.92 0.93 0.83	EIF2 signaling	13.6	1.21	*RPL11, RPLP1, SOS2, PDPK1, RPS11, BCL2, RPS20, UBA52, RPS13, EIF3A, IGF1R, RPS3, RPS5, RPL32, RPS19, NRAS, RALB, RPL29, RPS10, AGO2, RPS21, RPL23, RPL28, FAU, RPL10A, EIF3G, RPL8, RPS16, SREBF1, RPS27L, EIF4A1, EIF3I, RPS27A, RPL10, RPS25, RPS14, RPSA, EIF3K*
				Regulation of eIF4 and p70S6K signaling	8.83	−1.34	*SOS2, PDPK1, RPS11, RPS20, RPS13, EIF3A, RPS3, RPS5, ITGB1, RPS19, NRAS, RALB, RPS10, AGO2, RPS21, FAU, EIF3G, RPS16, RPS27L, EIF4A1, EIF3I, RPS27A, RPS25, RPSA, RPS14, EIF3K*
				Oxidative phosphorylation	5.01	4.0	*SDHA, NDUFV1, COX4I2, COX6A1, ATP5F1D, NDUFB8, MT-CO2, NDUFA2, ATP5F1B, UQCR10, NDUFB7, CYC1, COX5A, CYB5A, UQCRC1, UQCRQ*
				Mitochondrial dysfunction	7.37	NaN	*HSD17B10, SDHA, NDUFV1, COX4I2, ATP5F1D, COX6A1, PRDX5, TRAK1, BACE1, NDUFB8, MT-CO2, DHODH, GPX7, NDUFA2, BCL2, ATP5F1B, TXN2, UQCR10, CYC1,NDUFB7, COX5A, CYB5A, UQCRC1, ACO1, UQCRQ*

*^1^Number of genes (in the GNM) with known function. ^2^p < 0.01, r >±0.75.*

**FIGURE 5:**
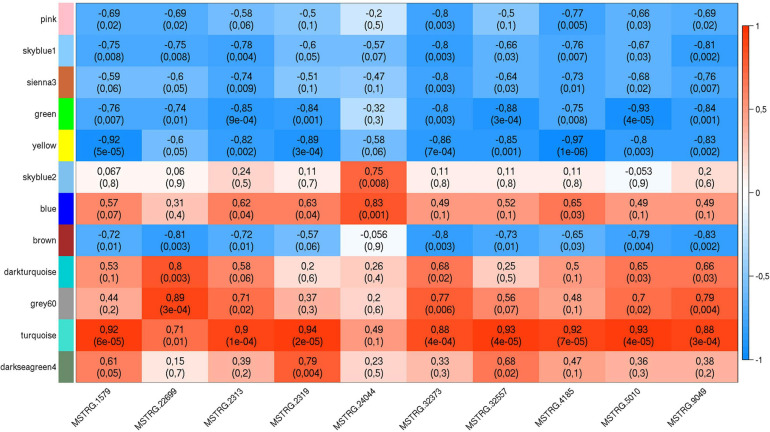
Weighted gene co-expression plot with co-expression blocks corresponding to the significantly co-expressed (*p* < 0.01) gene network modules (GNMs) (on the **left**) and the 10 selected DE lncRNAs (on the **bottom**). The left axis consists of color codes of each GNM, the right axis is the correlation coefficient (*r*) reference scale with integer value and color intensity (red to blue) adopted for each lncRNA and GNM block. Each block consists of correlation coefficient (on the **top**) and p-value (in brackets) corresponding to each GNM-lncRNA pair.

The co-expression and IPA analyses revealed that the *ERK5 Signaling*, *SAPK/JNK Signaling* and *RhoGDI Signaling* canonical pathways were enriched by genes included in the GNMs ‘green’ and ‘yellow’ (Table 2), which were both negatively correlated with expression levels of several DE lncRNAs (Table 2 and Figure 5). In addition, genes from GNM ‘turquoise’ that showed a positive correlation with DE lncRNA expression levels (Table 2 and Figure 5) were enriched in the canonical pathways *EIF2 Signaling* and *Regulation of eIF4 and p70S6K Signaling* (Table 2). It is striking that the same lncRNAs (MSTRG.1579, MSTRG.2313, MSTRG.2319, MSTRG.32373, MSTRG.32557, MSTRG.5010, MSTRG.9049, and MSTRG.4185) correlated with the GNMs ‘green,’ ‘yellow,’ and ‘turquoise’ indicating that they might be involved in the regulation of specific processes within the respective interconnected biological pathways, which display variation regarding resistance against GIN infection. An intersection between these pathways might be the mitogen-activated protein kinase (MAPK) pathway, which comprises the ERK, JNK and p38 mediated kinase cascades. It represents a conserved host-defense repertoire, and dysfunctions are known to result in hypersensitivity toward infection and stress (Johnson and Lapadat, 2002).

## Discussion

LncRNAs are known to play multiple biological functions and their expression also varies with the developmental stage of cells and tissues and under different disease states and environmental challenges (Ma et al., 2012). Thus, by inferring the functional role of lncRNAs we can attempt to better understand the possible mechanisms of complex biological processes related to various metabolic disorders, disease conditions, divergent phenotypes and response to environmental challenges. However, compared to regular protein-coding genes, the sequences and secondary structures of lncRNA transcripts are usually not conserved (Mercer et al., 2009; Pang et al., 2009). This makes it difficult to investigate the function of lncRNAs directly based on their physical nucleotide structure and features. The availability of different bioinformatics tools such as *CNCI* (Sun et al., 2013), *PLEK* (Li et al., 2014), *PLAR* (Hezroni et al., 2015), and *FEELnc* (Wucher et al., 2017) has enabled to predict lncRNAs from unknown transcripts, but the detection of their biological functions is still challenging. It has been reported that lncRNAs exert their functions by regulating or interacting with other molecules like RNA, DNA and proteins (Ma et al., 2012; Schmitz et al., 2016). Thus, one possible approach to predict the function of lncRNAs is to explore the relationship between lncRNAs and other molecular interacting partners. One such approach is to identify the protein-coding genes that are co-expressed with the interrogated lncRNA. According to the principle of “guilt by association,” the putative lncRNA function can be assigned via correlation to a group of co-expressed genes (in a module) with the biological pathway enriched by the group of co-expressed genes (Wolfe et al., 2005). This approach has been applied for a variety of lncRNA studies in other species (Petri et al., 2015; Li et al., 2016; Zhang et al., 2017; Weikard et al., 2018; Zheng et al., 2018; Ling et al., 2019; Nolte et al., 2019; Thiel et al., 2019; Wang et al., 2020).

In this context, the current study was performed to predict ovine lncRNA transcripts in the ALN transcriptome and to investigate the functional role of DE lncRNAs in respective samples obtained from adult sheep after an experimental GIN infection. This was carried out in several steps: In the first step, we adopted a pipeline using bioinformatics tools for the detection and classification of lncRNAs expressed in the transcriptome of the ovine ALN. In the second step, differential gene expression and subsequent gene co-expression analyses were performed to establish co-expression networks of transcripts in gene expression modules, GNMs, followed by a correlation analysis between the expression level of 10 selected DE lncRNA loci and GNMs. In the final step, the potential functional role of these 10 target lncRNAs was predicted indirectly by performing gene enrichment and canonical biological pathway analyses with the DE genes of the significantly correlated GNMs.

### Co-expression of LncRNAs With Genes From ERK5 and SAPK/JNK Signaling Pathways

The canonical pathway *ERK5 (Extracellular signal-related kinase) Signaling* (*z*-score = −2.5) (Supplementary Figure 4) was on top of the enriched pathways in the GNM ‘green.’ The involved signal transduction molecules are known to elicit several biological responses and to regulate cell functions such as tissue morphogenesis, cell proliferation, differentiation, migration and survival, apoptosis, cytoskeletal rearrangements, immune response and adaptation or stress response as reviewed by different authors (Dong et al., 2002; Drew et al., 2012; Arthur and Ley, 2013; Stecca and Rovida, 2019). The genes from the GNM ‘green,’ belonging to the *ERK5 Signaling* pathway, were *ELK4* (encoding a transcription factor involved in both transcriptional activation and repression) and *GNA13* (associated with PKA Signaling and Rho-related Signaling). They had a higher expression level in the ALN transcriptome of RES compared to SUS sheep in response to GIN challenge and were also found to be significantly higher expressed in susceptible goat (Bhuiyan et al., 2017) and in susceptible selection lines of Perendale sheep (Diez-Tascon et al., 2005), respectively, in response to GIN infection (Bhuiyan et al., 2017).

Pathway enrichment analysis of the GNM ‘green’ showed co-expression of lncRNAs with genes included in the *SAPK/JNK Signaling* pathway (*z*-score = −2.5). The stress-activated protein kinase/c-Jun N-terminal kinases (SAPK/JNK) are known to be affected by many types of cellular stresses and extracellular signals, such as UV irradiation, inflammatory cytokines and growth factors. They participate in numerous different intracellular signaling pathways that control cellular processes, including cell proliferation, differentiation, transformation and migration, cytoskeletal integrity and DNA repair (Nishina et al., 2004). Finally, the modulation of the SAPK/JNK pathway by stress stimuli results in transcriptional regulation of stress-related genes. In our study, the genes *MAP3K13* and *MAP3K7* of the MAPK family were included in the *SAPK/JNK Signaling* pathway (Table 2), and both were lower expressed in the ALN transcriptome of RES compared to SUS sheep. MAP3K7 is a key signaling component of nuclear factor-κB (NF-κB) and MAPK signaling pathways and acts as an essential regulator of innate immune signaling and apoptosis and of the proinflammatory signaling pathway. It also plays a central role in adaptive immunity in response to physical and chemical stresses [reviewed by Dai et al. (2012)]. MAP3K13 is able to activate JNK (Ikeda et al., 2001), is implicated in NF-κB activation (Ikeda et al., 2001; Masaki et al., 2003) and a role in a variety of developmental, stress-sensing, and disease contexts is assumed (Jin and Zheng, 2019).

### Co-expression of LncRNAs With Genes Acting in RhoGDI and Axonal Guidance Signaling Pathways

**FIGURE 6:**
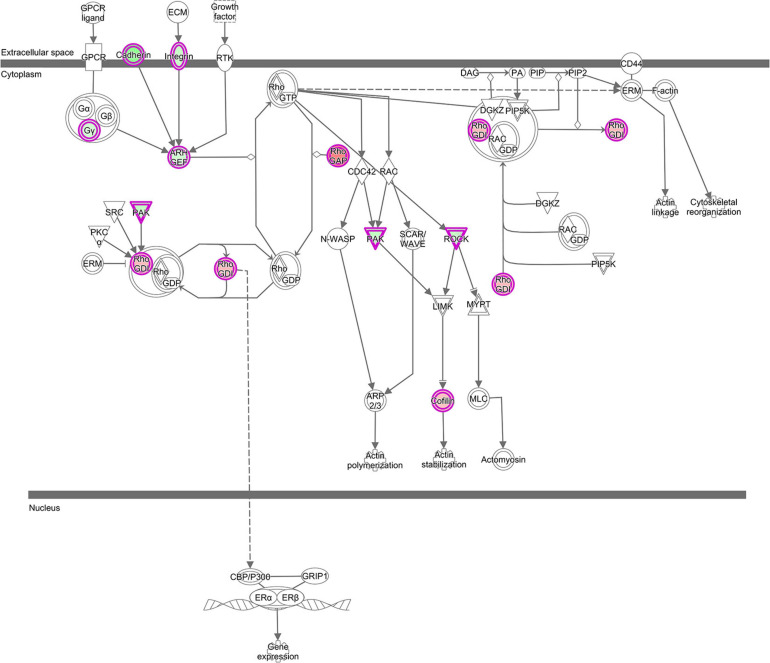
“RhoGDI Signaling canonical pathway” from IPA, was the top enriched pathway with genes from GNM ‘yellow’ with *z*-score = 2.33. This figure is adapted from Ingenuity Pathway Analysis^®^.

Stress signals are known to be delivered to the SAPK/JNK signaling cascade by small GTPases of the Rho family. Our analysis revealed that lncRNAs mentioned above were also co-expressed with genes involved in pathways, which are linked to GTPases of the Rho family: *RhoGDI (Rho-specific guanine nucleotide dissociation inhibitor) Signaling* (*z*-score = 2.33) and 4 *Actin-based Motility by Rho* (*z*-score = −1.0) (Supplementary Table 2 and Figure 6). These pathways all were enriched in the GNM ‘yellow.’ Rho GDP dissociation inhibitors (RhoGDIs) play important roles in various cellular processes, including cell migration, adhesion and proliferation, differentiation, cytoskeletal reorganization and membrane trafficking by regulating the functions of the Rho GTPase family members. Dissociation of Rho GTPases from RhoGDIs is necessary for their spatiotemporal activation (Dransart et al., 2005; Garcia-Mata et al., 2011; Cho et al., 2019). Several DE genes in the RhoGDI Signaling pathway, such as *ARHGEF10, DLC1* and *ARHGDIA*, were co-expressed with lncRNAs (Supplementary Table 2). The respective protein, ARHGEF10 is a guanine nucleotide exchange factor regulating activation of Rho GTPases, DLC1 belongs to the GTPase-activating proteins involved in the inactivation of Rho GTPases, whereas ARHGDIA is a RhoGDI, responsible for maintaining a stable pool of inactive Rho-GTPases (Stradal and Schelhaas, 2018). Co-expression of lncRNAs with the *ARHGDIA* gene that is higher expressed in the ALN transcriptome of RES compared to SUS sheep in our study might indicate a regulatory role of those lncRNAs for *ARHGDIA* expression. These lncRNAs were also co-expressed with ROCK kinase genes (*ROCK1* and *ROCK2*), which are included in all enriched Rho-associated pathways mentioned above but also in several *Ephrin Signaling* -related and *Axonal Guidance Signaling* pathways (Supplementary Table 2). In our study, both *ROCK* kinase isoform transcripts were lower expressed in the ALN transcriptome of RES compared to SUS sheep. Multiple functions of ROCK kinases have been detected in biological processes including cell contraction, migration, apoptosis, survival, and proliferation (Julian and Olson, 2014). The proteins are key regulators of actin organization, which link them to the actin-depolymerizing factor CFL1 that showed higher expression in the ALN transcriptome of RES versus SUS sheep in our study. CFL1 acts in Rho-induced reorganization of the actin cytoskeleton (actin depolymerisation/filament stabilization) and is known as key player in controlling the temporal and spatial pattern of actin dynamics, which is crucial for mediating host–pathogen interactions (Zheng et al., 2016). All intracellular and even some extracellular pathogens affect the host cell cytoskeleton to promote their own survival, replication, and dissemination (Stradal and Schelhaas, 2018). In summary, the RhoGDI canonical pathway also in conjunction with other Rho- related and Ephrin- related signaling cascades as well as with the *Axonal Guidance Signaling* pathway might be involved in controlling the differential resistance to nematode infection in sheep and possibly, all of these processes may be modulated by co-expressed lncRNAs.

### Co-expression of LncRNAs With Genes Involved in Protein Biosynthesis, Apoptosis and Mitochondrial Function Pathways

Furthermore, the co-expression analysis revealed that these lncRNAs were also connected with genes present in the GNM ‘turquoise’ suggesting a functional role of the canonical pathways *EIF2 Signaling* (*z*-score = 1.21) in conjunction with *Regulation of eIF4 and p70S6K Signaling* and *mTOR Signaling* (*z*-score = −1.34 for both) in sheep characterized by differential resistance to nematode challenge. The respective signaling pathways are engaged in the control of protein biosynthesis by regulating the translational machinery at different cascade steps (e.g., Supplementary Figures 5, 6). As translation is a tightly regulated process in response to various stimuli, including extracellular and intracellular signals and environmental stress conditions, translational control plays a major role in host stress responses, including pathogenic infection and defense by enabling rapid responses to the challenge (Mohr and Sonenberg, 2012). In our study, the participation of these biological pathways in the modulation of differential resistance to GIN challenge is supported by the upregulated expression of numerous ribosomal protein genes, and translation initiation factors, which are known to act in the ribosome biogenesis and the mRNA translational machinery. These genes from the GNM ‘turquoise’ showed a higher expression in the ALN transcriptome of sheep with a superior resistance against GIN. A crucial signaling mediator is the *PDPK1* gene (*3-phosphoinositide dependent protein kinase 1*) that is involved in all three canonical pathways *EIF2 Signaling*, *Regulation of eIF4 and p70S6K Signaling* and *mTOR Signaling.* The encoding protein PDK1 is a master kinase, able to control numerous physiological and pathological processes (Di Blasio et al., 2017). In our study, *PDPK1* had a lower expression level in the ALN transcriptome of SUS sheep compared to the RES group. But in goats, *PDPK1* was significantly higher expressed in susceptible animals after GIN infection (Bhuiyan et al., 2017).

GNM ‘turquoise’ also contained the antiapoptotic *BCL2* gene in the gene set associated with EIF2 signaling. The encoded BCL2 protein is known to be tightly connected with cellular survival. It suppresses apoptosis in many cell systems and regulates cell death by controlling the mitochondrial membrane permeability (Youle and Strasser, 2008; Siddiqui et al., 2015). Apoptosis, a mechanism by which cells infected with pathogens can be eliminated without triggering an unwanted inflammatory response, seemed to be upregulated in the RES group in response to GIN challenge in our study, which is supported by the higher *BCL2* expression in the RES compared to the SUS sheep. In the study of Ingham et al. (2008), the *BCL2* gene was quantified in the mucosa of the GIN tract of lambs of resistant and susceptible lines subjected to of *Haemonchus contortus* and *Trichostrongylus colubriformis* challenge, and it was identified as one of the candidate genes for maintaining the epithelial integrity of the gut in response to GIN. Furthermore, the *BCL2* gene was higher expressed in the duodenum of in Naïve Perendale sheep genetically susceptible to GIN compared to resistant animals (Keane et al., 2006), which is in line with our observations.

A regulatory link of these pathways is suggested due to the simultaneous co-expression with several lncRNAs that were also upregulated in the ALN transcriptome of those sheep characterized by a better resistance to nematode challenge. The functional interplay between protein biosynthesis cascades and PI3K/AKT- and MAPK- related pathways is clearly illustrated by the intertwining of the GNM ‘turquoise’ with the GNMs ‘green’ and ‘yellow’ and the associated lncRNAs. A closer look at the genomic location of the correlated lncRNAs revealed that some of them (MSTRG.9049, MSTRG.32373, MSTRG.2313, and MSTRG.5010) are located in genomic regions near ribosomal protein genes, which would indicate a regulatory potential for them to control the expression of the respective neighbored genes via *cis* -regulation. Considering that host cells respond to stress and imbalances by modifying gene expression at epigenetic, transcriptional and translational levels for recovering from the pathogen attacks, Knap et al. (2017) reviewed the state of knowledge on whether host cells would deploy lncRNAs to rapidly control host translation, the most energy-consuming process in cells, to counteract infection. The authors noted that the cause-effect relationship between the expression of lncRNAs and the activation of signaling pathways that control translation is currently unclear, but it is tempting to speculate that host cells could use this class of ncRNAs to fine-tune translation and cope with the imbalances triggered by pathogens.

Interestingly, numerous genes contained in the GNM ‘turquoise’ indicated an association of the correlated lncRNAs with the intertwined canonical pathways *Oxidative Phosphorylation* (*z* – score = 2.45) and *Mitochondrial Dysfunction*. These genes encode for various protein components of all complexes forming the mitochondrial electron transport chain, which drives oxidative phosphorylation and appeared to be upregulated in the ALN transcriptome of the sheep group with a better resistance to GIN challenge (Supplementary Figure 7). Mitochondria are the key organelles of energy production and coordinate essential metabolic processes in the cells. In addition to their central role in metabolism, mitochondria also regulate cellular processes such as cell cycle, innate immunity and apoptosis [summarized by Mills et al. (2017) and Mohanty et al. (2019)]. Recent findings highlight the emerging role of mitochondria as important intracellular signaling platform that regulates innate immune and inflammatory responses (Jin et al., 2017). LncRNAs have been reported to be involved in regulating mitochondrial processes such as mitochondrial respiration, reactive oxygen production and apoptosis (De Paepe et al., 2018) and are hypothesized to coordinate functions between mitochondria and the nucleus (Dong et al., 2017). To maintain homeostasis of the cells, an intense cross-talk between mitochondria and the nucleus, mediated by lncRNAs is conceivable. Thus, lncRNAs that were highly correlated to genes of the GNM ‘turquoise’ might be associated with the regulation of mitochondrial bioenergetics and biosynthesis. Taken together, of the 10 DE lncRNAs selected for co-expression analysis, eight were found to be potentially involved in the regulation of these biological pathways with relevance to GIN infection, namely MSTRG.1579, MSTRG.2313, MSTRG.2319, MSTRG.32373, MSTRG.32557, MSTRG.5010, MSTRG.9049, and MSTRG.4185 (Table 2). Thus, these lncRNAs may contribute to the divergent resistance to gastrointestinal parasites.

### Potential Functional Genomic Interaction Partners of Target LncRNAs Potentially Involved in Regulating Divergent Resistance to Gastrointestinal Parasites in Sheep

LncRNAs might activate or repress the transcription of nearby genes (*cis*-regulation) present on the same (Engreitz et al., 2016) or opposite strand (Villegas and Zaphiropoulos, 2015; Tan-Wong et al., 2019).

As already mentioned, MSTRG.9049, MSTRG.32373, MSTRG.2313, and MSTRG.5010 are located in genomic regions antisense to DE ribosomal protein genes, *RPS19, RPS20*, and *RPL35A, RPL23* indicating a potential functional regulatory link to translation-associated processes.

MSTRG.1579 is overlapping with a known lincRNA (*ENSOARG00000025559*), and the *TAGLN2* gene region. The *TAGLN2* is an actin binding protein and regulates the T cell activation in mammals by stabilizing the actin cytoskeleton (Na and Jun, 2015).

MSTRG.2319 represents a novel, not yet annotated intergenic lncRNA localized between the genes *MUC20* and *TNK2*, which however, were not DE between the different GIN-resistance groups. *MUC20* encodes a member of the mucin glycoprotein family implicated in protection of all mucosal surfaces. Membrane bound mucins have been suggested to play a functional role in cell signaling linked to health and disease (Corfield et al., 2001; Linden et al., 2008). A *MUC20* -lncRNA has been reported to bind ROCK1 and to be functionally involved in tumor suppression (Dai et al., 2020) indicating *trans*-regulation. *TNK2* encodes a tyrosine kinase that binds to the Rho family member Cdc42Hs and inhibits both the intrinsic and GTPase-activating protein-stimulated GTPase activity (Manser et al., 1993). Thus, MSTRG.2319 could be primarily involved in the pathway Signaling by Rho Family GTPases.

The lncRNA MSTRG.32557 is located near the *FABP4* gene region, which encodes a member of the fatty acid binding protein family that plays a role in lipid metabolism by binding and intracellular transport of long-chain fatty acids. *FABP4* showed a higher expression level in the ALN transcriptome of RES sheep and was found to be DE between RES and SUS sheep groups in our previous transcriptome study performed with the same tissue and conditions (Chitneedi et al., 2018). Other studies also imply roles of FABP family proteins in cell signaling, inhibition of cell growth and cellular differentiation. Furthermore, FABP also modulates tumor cell growth, metabolism, migration, differentiation and development involving the PI3K/AKT signaling and PPAR-associated pathways (Amiri et al., 2018). Specifically, FABP4 has been found to function as an adipokine that is involved in regulating macrophage and adipocyte interactions during inflammation (Thumser et al., 2014). A GWAS study showed that FABP4 contributes to resistance to fleece rot in Australian merino sheep (Smith et al., 2010). This gene was also found to be downregulated in response to GIN *Cooperia oncophora* infection in cattle (Li and Schroeder, 2012).

The lncRNA MSTRG.4185 is located close to the chemokine (C-C motif) ligand 14 *(CCL14)* gene region. The cytokine encoded by this gene induces changes in intracellular calcium concentration and enzyme release in monocytes and showed strong correlation with tumor immune cells infiltration (Gu et al., 2020). The expression of *CCL14* along with other chemokine ligands was reported to be significantly higher in a resistance sheep flock compared to a susceptible flock after *Haemonchus contortus* infection (Zhang et al., 2019). As the *CCL14* gene was not DE between RES and SUS sheep groups in our study, but MSTRG.4185 was highly correlated with GNMs (most negatively to the ‘yellow’ GNM and also positively correlated to the ‘turquoise’ GNM, Figure 4), a putative *trans*-regulation of genes included in correlated biological pathways could be assumed.

The lncRNAs, which are co-expressed with the GNMs ‘green,’ ‘yellow,’ and ‘turquoise,’ could possibly be involved in the regulation of the expression of genes included in the respective network modules. These modules, in turn are involved in pathways associated with different physiological and environmental conditions as well as with divergent phenotypic characteristics, such as the modulation of a differential resistance or susceptibility of adult sheep to parasite infection. Results obtained from this study only provide first hints on the genes and pathways that are primarily targeted by individual lncRNAs under interrogation. As this is an initial study, further research in sheep in response to GIN infection will be required to establish the functional role of the detected lncRNA transcripts.

Overall, this preliminary lncRNA study, conducted in adult sheep after GIN infection gave first insights into the potential functional role of selected lncRNAs by investigating their putative functions via co-expressed genes based on the principle of “guilt-by-association.” Future multi-omics studies including DNA, RNA and metabolites will help to gain a better understanding of the general and specific roles of the selected lncRNAs and other lncRNAs significantly involved in the regulation of key physiological pathways associated with resistance against GIN infection in sheep.

## Data Availability Statement

The 12 abomasal lymph node raw RNA-Seq datasets analyzed in this study have been submitted to the ENA Read Archive at EMBL-EBI following FAANG DCC guidelines. They are available at https://www.ebi.ac.uk/ena/browser/view/PRJEB33 476 (Bioproject PRJEB33476). Data on the meta data of the samples are submitted as https://www.ebi.ac.uk/biosamples/samples/SAMEG4750853.

## Ethics Statement

The animal study was reviewed and approved by the subcommittee for experimentation and animal welfare (OBEA) of the University of León and the competent body of the Junta de Castilla y León regional government (Spain) (Ref. ULE_024_2015).

## Author Contributions

BG-G and JA conceived and designed the study, and performed the collection and processing of tissue samples. MM-V designed and performed the experimental infections. BG-G, JA, and MM-V performed sampling in the commercial flock and selected the animals to be experimentally infected. CK and RW optimized the bioinformatics workflow. PC performed the bioinformatics analyses under the supervision of CK. PC and RW wrote the manuscript. BG-G, CK, and JA critically reviewed the manuscript. All authors read and approved the final manuscript.

## Conflict of Interest

The authors declare that the research was conducted in the absence of any commercial or financial relationships that could be construed as a potential conflict of interest.

## Footnotes

1 http://www.ensembl.org/Ovis_aries_rambouillet/Info/Strains?db=core

2 http://www.ensembl.org/Ovis_aries_rambouillet/Info/Index

3 http://ftp.ensembl.org/pub/release-95/gtf/ovis_aries/

4 https://digitalinsights.qiagen.com/products-overview/discovery-insights-portfolio/analysis-and-visualization/qiagen-ipa/

## Supplementary Material

Supplementary Figure 1 Bioinformatics workflow showing the different steps performed to detect long non-coding RNAs (lncRNAs) in the abomasal lymph node transcriptome from resistant or susceptible sheep to GIN infection and to predict their potential functional role by gene co-expression and pathway enrichment analysis.

Supplementary Figure 2 Distribution of accumulated fecal egg count (FEC) in resistant and susceptible sheep after a second experimental infection with GIN *T. circumcincta.* Blue bars represent the FEC values in each resistant sample and yellow bars represent the FEC values in each susceptible sample. **(B)** PCA performed with the read count matrix of ALN RNA-seq datasets using R function “varianceStabilizingTransformation” by specifying the sample condition (resistant and susceptible) with the initially considered 6 resistant and 6 susceptible samples.

Supplementary Figure 3 Weighted gene co-expression network matrix plot. Each block corresponds to the co-expression result of each gene network module (GNM, **left**) and a lncRNA (bottom). The left axis consists of color codes for each GNM and the right axis is the correlation coefficient (*r*) reference scale with integer values and color intensity (red to blue) adopted for each lncRNA and GNM block. Each block consists of the correlation coefficient **(top)** and *p*-value **(bottom)** corresponding to each GNM-lncRNA pair.

Supplementary Figure 4 “EIF2 Signaling” from IPA, was the top enriched pathway with genes from the GNM ‘turquoise’ with *z*-score = 1.21. This figure is adapted from Ingenuity Pathway Analysis^®^.

Supplementary Figure 5 “ERK5 Signaling” from IPA, was the top enriched pathway with genes from the GNM ‘green’ with *z*-score = −2.5. This figure is adapted from Ingenuity Pathway Analysis^®^.

Supplementary Figure 6 “Regulation of eIF4 and p70S6K Signaling” from IPA, was the second top enriched pathway with genes from the GNM ‘turquoise’ with *z*-score = −1.34. This figure is adapted from Ingenuity Pathway Analysis^®^.

Supplementary Figure 7 “Oxidative Phosphorylation” from IPA, was one of the top enriched pathways with genes from the GNM ‘turquoise’ with *z*-score = 4.00. This figure is adapted from Ingenuity Pathway Analysis^®^.

Supplementary Table 1 Differentially expressed gene loci sorted based on p-value and the list of associated long non-coding RNAs (lncRNAs) along with the information of novel lncRNAs.

Supplementary Table 2 List of top 20 pathways based on −log (*p*-value) enriched by the genes from the three GNMs green, yellow and turquoise according to the Ingenuity Pathway Analysis^®^.

Supplementary File 1 Bash script with all the parameters adapted for different tools used in the bioinformatics workflow of lncRNA detection.
