# Supplementary figures and images for: Identification of Regulatory Functions of LncRNAs Associated With T. circumcincta Infection in Adult Sheep
Source: Front Genet. 2021 Jun 14;12:685341. doi: 10.3389/fgene.2021.685341 (PMC8236958; doi:10.3389/fgene.2021.685341)

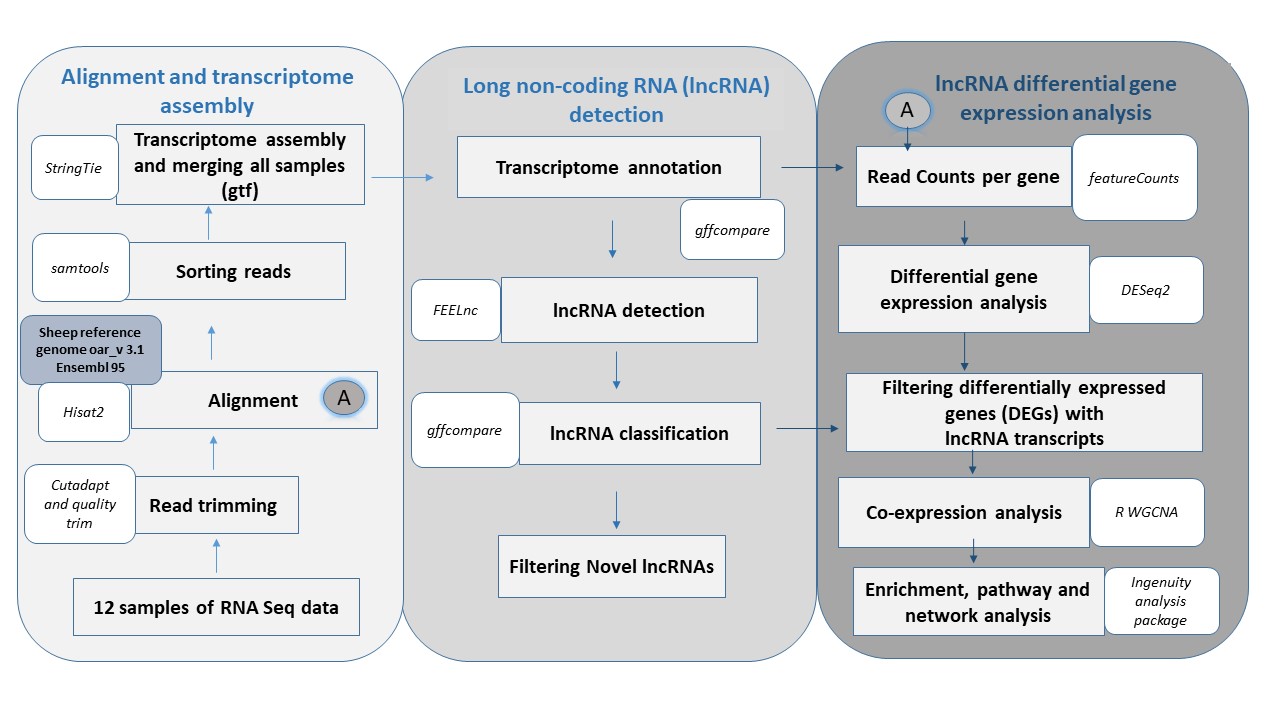

Supplement: Supplementary Figure 1 — Bioinformatics workflow showing the different steps performed to detect long non-coding RNAs (lncRNAs) in the abomasal lymph node transcriptome from resistant or susceptible sheep to GIN infection and to predict their potential functional role by gene co-expression and pathway enrichment analysis. [file Image_1.JPEG]

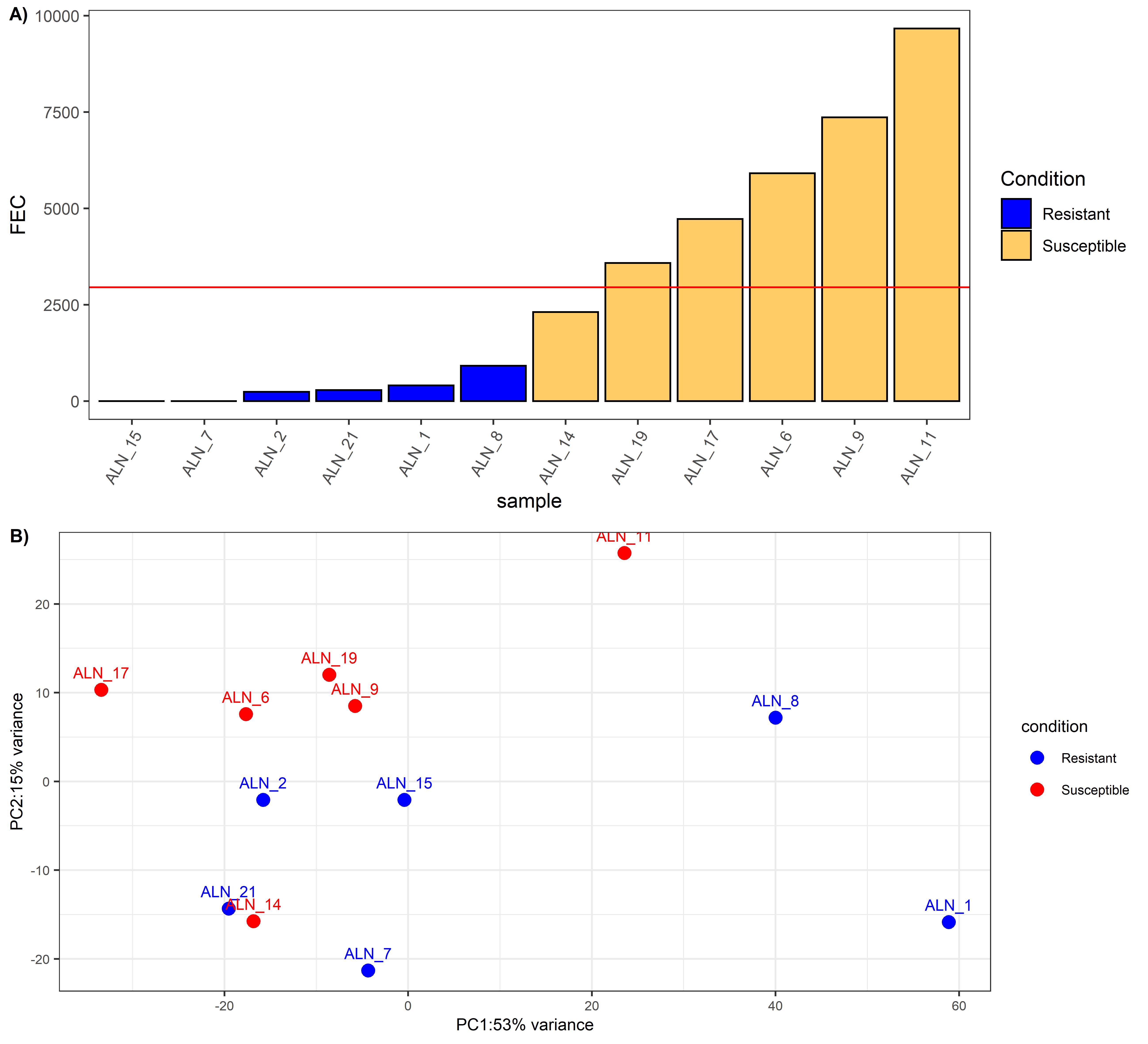

Supplement: Supplementary Figure 2 — Distribution of accumulated fecal egg count (FEC) in resistant and susceptible sheep after a second experimental infection with GIN T. circumcincta. Blue bars represent the FEC values in each resistant sample and yellow bars represent the FEC values in each susceptible sample. (B) PCA performed with the read count matrix of ALN RNA-seq datasets using R function “varianceStabilizingTransformation” by specifying the sample condition (resistant and susceptible) with the initially considered 6 resistant and 6 susceptible samples. [file Image_2.JPEG]

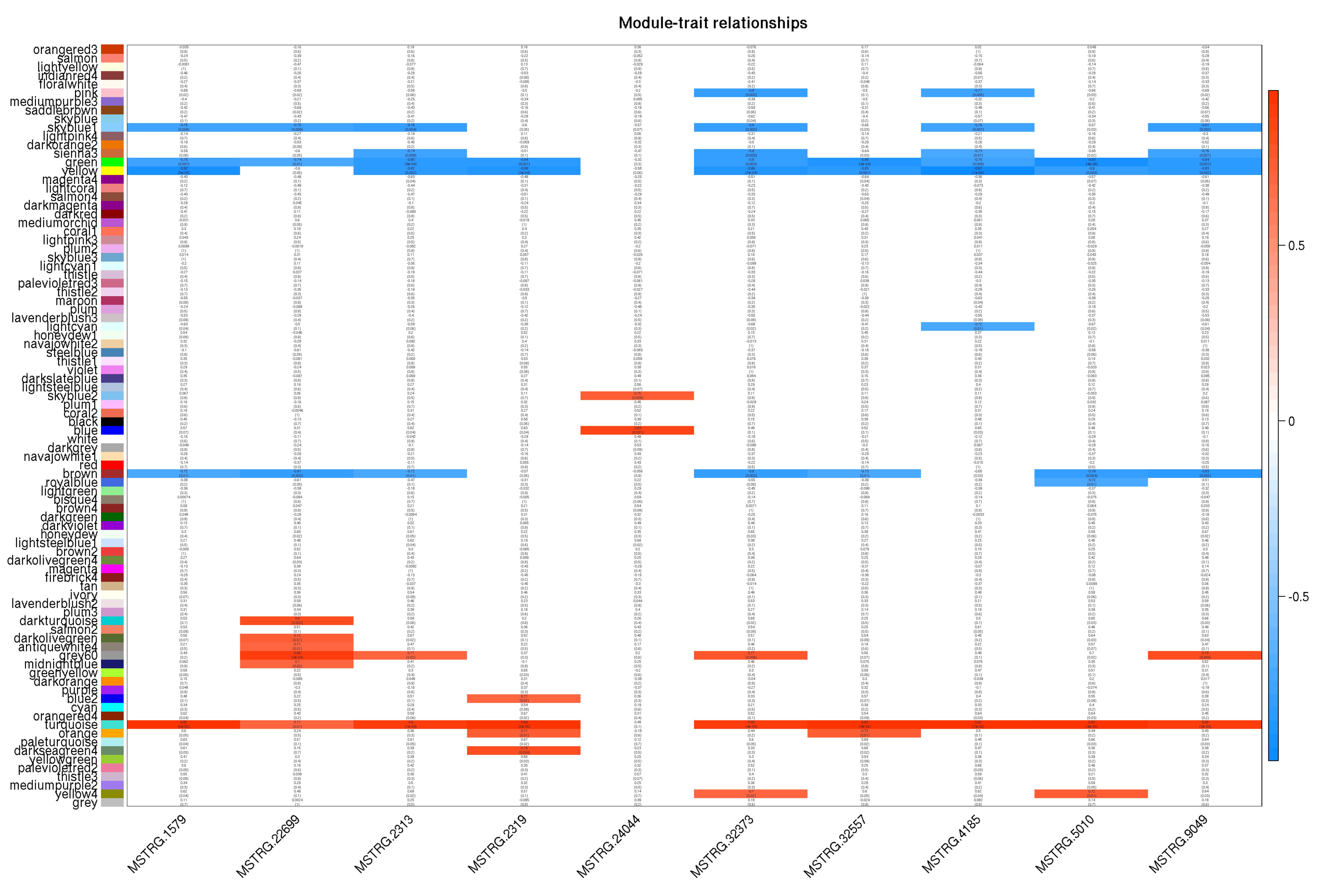

Supplement: Supplementary Figure 3 — Weighted gene co-expression network matrix plot. Each block corresponds to the co-expression result of each gene network module (GNM, left) and a lncRNA (bottom). The left axis consists of color codes for each GNM and the right axis is the correlation coefficient (r) reference scale with integer values and color intensity (red to blue) adopted for each lncRNA and GNM block. Each block consists of the correlation coefficient (top) and p-value (bottom) corresponding to each GNM-lncRNA pair. [file Image_3.JPEG]

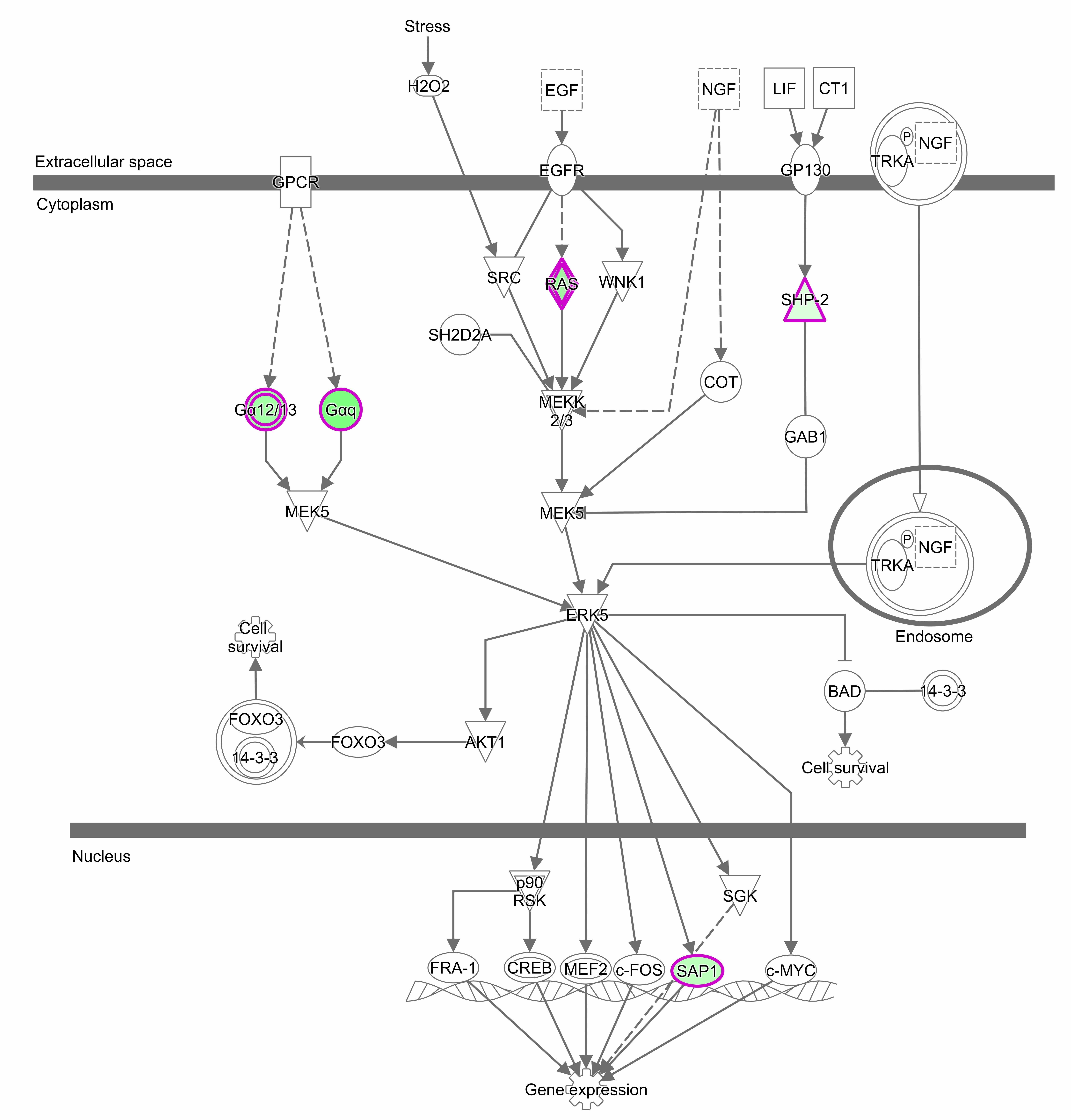

Supplement: Supplementary Figure 4 — “EIF2 Signaling” from IPA, was the top enriched pathway with genes from the GNM ‘turquoise’ with z-score = 1.21. This figure is adapted from Ingenuity Pathway Analysis®. [file Image_4.JPEG]
